# Further investigation of the impact of foveal and parafoveal word frequency on parafoveal preview during Chinese reading

**DOI:** 10.1371/journal.pone.0340103

**Published:** 2026-01-20

**Authors:** Yue Sun, Sainan Li, Yancui Zhang, Jingxin Wang

**Affiliations:** 1 Faculty of Psychology, Tianjin Normal University, Tianjin, China; 2 School of Educational Sciences, Heihe University, Heilongjiang, China; 3 Tianjin Academy of Educational Sciences, Tianjin, China; 4 College of Humanities, Tianjin Agricultural University, Tianjin, China; Hong Kong Baptist University, HONG KONG

## Abstract

Parafoveal preview is crucial for cognitive language processing, significantly improving reading efficiency. However, the factors influencing this process remain ambiguous. Given the unique characteristics of Chinese, it is unclear whether the processing load of foveal words, parafoveal words, or a combination of both impacts parafoveal preview. To investigate these issues, we employed eye-tracking technology and manipulated the frequency of both foveal and parafoveal words, as well as two types of preview. Sixty-four valid participants participated in the study, with two-character words serving as foveal and parafoveal stimuli. The results indicated that the frequency of foveal words affected the preview benefit: as the foveal load increased, the preview benefit decreased, demonstrating the foveal load effect. However, parafoveal word frequency had no effect on the preview benefit, nor was there any interaction between foveal and parafoveal word frequency in influencing the preview benefit. Additionally, both foveal and parafoveal processing loads independently affected the forward saccade length: lower foveal processing load resulted in longer forward saccades, while greater parafoveal preview availability also increased saccade length. These findings support the Chinese Reading Model (CRM) and provide valuable empirical evidence for refinement.

## Introduction

Reading is widely regarded as a fundamental activity essential to daily life. Although reading may seem simple, it involves a complex cognitive process, making studying this process highly significant. Reading is inseparable from eye movements. Eye-tracking technology enables researchers to monitor readers’ eye movements in real-time, thereby facilitating an investigation of the cognitive processes underlying reading through appropriate experimental design.

During reading, individuals extract information not only from the fovea, the area of the visual field currently being fixated but also from the parafovea, the adjacent region that remains unfocused. Parafoveal word preprocessing, known as parafoveal preview, has been widely shown to be crucial in guiding saccade target selection and facilitating parafoveal word recognition [1–4]. As a result, parafoveal preview significantly enhances reading efficiency and reduces processing time. The resulting reduction in processing time is called the parafoveal preview benefit [2–4]. Parafoveal preview is typically investigated using the boundary paradigm [4–5], which measures the extent of preview processing by manipulating the availability of valid parafoveal information. The preview benefit is quantified as the difference in processing time between conditions with valid and invalid parafoveal preview.

Studies on alphabetic languages have shown that both the processing load of foveal words [6–10] and parafoveal words [1,4,11] can influence the parafoveal preview. In two experiments, using word frequency and syntactic complexity as the foveal load, Henderson et al. (1990) showed that increased foveal load diminished the preview benefit. This phenomenon, termed the foveal load effect, refers to the impact of foveal load on parafoveal preview. As a core effect in reading, the foveal load effect is integrated into the most prominent eye movement control models for alphabetic languages, such as the E-Z Reader model [12,13] and the SWIFT model [14–16]. However, subsequent studies have yielded mixed results replicating Henderson’s findings [17–23]. Research on alphabetic languages has primarily focused on how awareness of the “unnatural” baseline used in the boundary paradigm affects the foveal load effect; however, no consensus has been reached [18,21–23]. Some researchers also have suggested that the inconsistent results on alphabetic languages are due to the high variability in word lengths, especially the length of parafoveal words [24–27]. Another factor affecting preview benefit is the parafoveal load. The lexical characteristics of parafoveal target words (e.g., word frequency and predictability) can affect parafoveal preview [1,4,11]. Inhoff et al. (1989) demonstrated that easier parafoveal processing (high-frequency words) enhances preview benefit. Additionally, foveal and parafoveal processing load may jointly influence parafoveal preview. Prior research has indicated that the foveal load effect is modulated by the difficulty of parafoveal processing, manifesting when the parafoveal word is challenging to process [10,19].

Cognitive processing in different languages shares commonalities but is also influenced by specific linguistic characteristics [28]. Chinese, a character-based language, features consistent character spacing and a concentrated distribution of word lengths, with 72% of words being two characters long and 6% being single characters. This allows for effective manipulation and control of word length. Additionally, unlike alphabetic scripts, Chinese lacks spaces between words and is more compact than alphabetic scripts, providing higher information density and enabling more parafoveal processing [29–31]. Moreover, Chinese lacks physical cues for word segmentation, which presents a unique challenge. Word segmentation is crucial for recognition during Chinese reading, as words are only recognized while segmentation is completed [28,32,33]. Given these characteristics, in Chinese reading, the factors influencing parafoveal preview may be distinct from those in alphabetic scripts. It is essential to investigate what factors affect the preview in Chinese reading.

Few studies have investigated the influence of foveal load on parafoveal preview in Chinese, and the findings are inconsistent. In Zhang and colleagues’ 2019 study, they manipulated the frequency of foveal words and two preview types (identical preview and pseudocharacter preview). However, they did not identify evidence of a foveal load effect. Building on this, Zhang et al. (2020a) replicated the experiment with the additional variable of reading speed; however, the results still failed to show the effect. Sun et al. (2024) [34] manipulated the frequency of both foveal and parafoveal words, as well as two preview types (identical preview and pseudocharacter preview). Their findings align with Zhang et al.’s (2019, 2020a) findings, showing no evidence of the foveal load effect. Notably, all three experiments used single-character words in the parafoveal region. Wang and Hu (2020) [35] manipulated the frequency of foveal words along with three preview types (identical preview, similar non-word preview, and dissimilar non-word preview). However, they differed in manipulating two-character words in the parafoveal region. Their results followed those previously reported by Henderson et al. (1990), which indicated the presence of the foveal load effect. Similarly, Yan (2015) [36] employed two-character words in the parafoveal region and manipulated visual complexity as the foveal load, along with three preview types (identical preview, unrelated word preview, and non-word preview). The study observed the foveal load effect, though in an inverse direction, potentially attributable to differing foveal load manipulations. Consequently, the inconsistency of the foveal load effect in Chinese reading may also be attributed to the length of the parafoveal words. Recently, Li and Pollatsek (2020) proposed a Chinese reading model (CRM) based on the interactive activation model, which simulates the foveal load effect, using word frequency as the foveal load. However, explaining the inconsistency of the foveal load effect remains challenging. In conclusion, research on the foveal load effect in Chinese is limited, and few studies have examined its underlying mechanisms. Further investigation into these mechanisms is needed to refine the CRM.

As mentioned above, in studies of alphabetic languages, the processing load of the parafoveal words affects the preview benefit. However, the results differ in Chinese reading. Wang et al. (2022) [37] manipulated character frequency and preview types (identical preview, pseudocharacter preview) for the first character of low-frequency two-character parafoveal words. This manipulation aimed to examine how parafoveal character frequency influences the preview. The results showed that although character frequency affected fixation durations on the parafoveal word, it had no effect on the preview benefit. Similarly, Wang et al. (2019) [38] investigated the impact of parafoveal word frequency on preview by manipulating the word frequency of the two-character parafoveal words along with the preview types (identical preview, pseudocharacter preview) for the first character of the two-character words. Their results demonstrated no effect of parafoveal word frequency on preview benefit. However, an interaction was observed on the length of the forward saccade. They proposed that the impact of parafoveal word frequency on preview is more closely associated with the planning of saccadic target selection than with preview benefit.

Furthermore, as previously mentioned, Sun et al. (2024) manipulated word frequency in the foveal and parafoveal regions, along with two types of preview; however, they did not find evidence of the foveal load effect or any other significant interactions. Their study focused on single-character words in the parafoveal region, which limits exploring these interactions. First, the majority of words in Chinese are two-character words (72%) and represent the typical case in Chinese reading. Second, previous research suggests that the length of parafoveal words may influence the foveal load effect, potentially affecting the three-way interaction. Third, due to the distinct features of the Chinese language, the use of single-character words makes it difficult to determine whether processing is influenced by character or word frequency. Therefore, further research examining the frequency of two-character words is warranted.

Therefore, the present study employed two-character words in the parafoveal region to examine the following questions. Firstly, does the frequency of foveal words affect preview benefit when parafoveal words consist of two characters? If the foveal load effect emerges, it may suggest a difference in the foveal load effect between single-character and two-character parafoveal words. What factors might account for this difference? Secondly, whether the frequency of words in the parafovea affects the preview benefit. Finally, it would be interesting to know whether the frequency of words in both the foveal and parafoveal regions collectively impacts the preview benefit. In particular, it would be valuable to determine whether parafoveal load modulates the foveal load effect, which tends to emerge when the parafoveal word is difficult to process, as demonstrated by previous studies in alphabetic languages [10,19].

In addition, if a foveal load effect is observed in this study, it would contrast with the results of Sun et al. (2024). Beyond differences in word length, single-character and two-character words may also differ in processing difficulty, with the two-character words being more challenging to process [31]. Thus, if these differences stem from parafoveal processing difficulty, manipulating this factor could reveal its modulatory influence on the foveal load effect, resulting in a three-way interaction. Conversely, if no such interaction is observed, the differences are more likely attributable to the spatial characteristics of word length rather than processing difficulty.

To investigate these questions, three factors were manipulated: the frequency of the foveal word, the frequency of the parafoveal word, as well as two types of preview. To examine whether the preview benefit of a two-character word is affected by the processing load of parafoveal words, this study employed the preview of the complete two-character words. Additionally, to minimize interference from parafoveal masking, orthographically legal pseudocharacters were employed as the baseline, differing from the approach taken by Wang and Hu (2020).

The present experiment postulated that when parafoveal words consist of two characters, foveal word frequency might exert an influence on preview benefit, thereby demonstrating the foveal load effect. Additionally, it was hypothesized that the frequency of parafoveal words may affect saccadic target selection planning rather than preview benefit. Moreover, a three-way interaction may exist, where the foveal load effect is observed only when parafoveal processing is difficult(low-frequency words). If such a three-way interaction is observed, the parafoveal processing load modulates the foveal load effect, indicating that differences in the foveal load effect between single-character and two-character parafoveal words stem from the processing difficulty of parafoveal words. Conversely, the absence of such an interaction would imply that the parafoveal processing load does not influence the foveal load effect, suggesting that these differences are more likely attributable to intrinsic word length properties rather than processing difficulty.

## Methods

### Participants

The number of participants was calculated using the MorePower 6.0.4 software following the methodology previously employed by Sun et al. (2024). Consequently, 67 students from the university participated, and three participants who were aware of the majority of displayed changes were excluded. The remaining 64 participants (male = 11; female = 53) were statistically analyzed, with an average age of 22.1 years (*SD* = 2.06).

The Research Ethics Committee of the Faculty of Psychology at Tianjin Normal University approved this study (Approval Number: 2024011201), which was conducted according to the principles outlined in the Declaration of Helsinki. All participants voluntarily participated in the experiment and provided written informed consent.

### Design and materials

**Fig 1:**
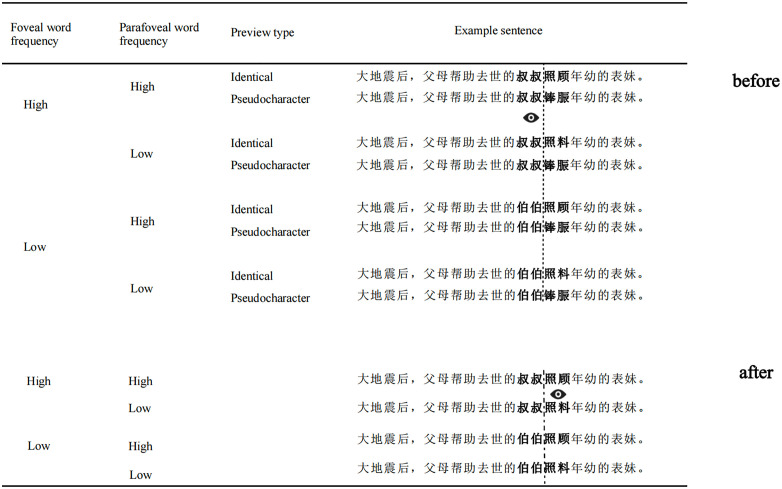
An example of experimental material. (Translation: After the great earthquake, my parents took over my deceased uncle’s responsibility to take care of my cousin). The invisible boundary is indicated by a dotted line, and the two-character words in bold preceding the dotted line are the foveal words (“叔叔/伯伯” means “uncle”), while those following the dotted line are the parafoveal words (“照顾/照料” means “take care of”). Prior to the eyes (“
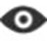
”) cross the boundary, the target word (“照顾/照料”) is substituted with a preview word (identical preview “照顾/照料” or pseudocharacter “
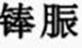
”), and following this, the preview word becomes the target word (“照顾/照料”).The pseudocharacter preview did not provide any valid information about the target word, resulting in longer processing times compared to the identical preview. This difference in processing time represents the preview benefit.

Eighty high- and low-frequency two-character word pairs were selected from the word frequency corpus [39] as the foveal words, and another eighty high- and low-frequency two-character word pairs as the parafoveal words, all within the same sentence frame. Significant differences in word frequency were observed for foveal word pairs (high-frequency: 48.26–11080.02/million, low-frequency: 0.09–6.80/million, *F* (1, 158) = 6.21, *p* = 0.01) and for parafoveal word pairs (high-frequency: 45.19–2360.84/million, low-frequency: 0.06–14.70/million, *F* (1, 158) = 37.89, *p* < 0.001). The number of strokes and the predictability of foveal and parafoveal word pairs were matched, showing no significant differences (*p*s > 0.09). All sentences had high fluency ratings (7 points) and were matched (*F*(3, 316) = 1.98, *p* = 0.12). The basic information is shown in Table 1. The experiment employed the boundary paradigm [4,5], as illustrated in Fig 1.

**Table 1:** The information of sentences.

	Foveal word frequency (per million)	Foveal word stroke	Foveal word predictability (%)	Parafoveal word frequency (per million)	Parafoveal word stroke	Parafoveal word predictability (%)	Sentence naturalness
High-frequency	344.85 (1230)	15.46 (4)	0.02 (.05)	224.69 (320)	15.53 (4)	HH: 0.02(.05)	HH: 5.40(.68)
						HL:0.01(.04)	HL:5.15(.78)
Low-frequency	2.22 (2)	15.91 (4)	0.01 (.05)	4.26 (4)	16.64 (4)	LH:0.02(.05)	LH:5.36(.77)
						LL:0.02(.04)	LL:5.23(.66)

The first “H/L” stands for foveal word frequency, and the second “H/L” stands for parafoveal word frequency.

#### Stimuli norming.

To control contextual predictability, cloze predictability tests were conducted separately for foveal and parafoveal target words. For the foveal words, 23 participants who did not take part in the eye-tracking experiment were presented with sentence fragments up to, but not including, the foveal word and were asked to complete the sentence with the first continuation that came to mind. The results indicated that both high- and low-frequency foveal words were low predictable, with no significant difference between frequency conditions. A separate cloze test was conducted for the parafoveal words. Participants were divided into two counterbalanced lists according to the frequency of the foveal word, with 16 participants assigned to each list. Parafoveal words were also low predictable across all four conditions, and no significant differences were observed. In addition, sentence naturalness was assessed by an independent group of 32 participants using a 7-point Likert scale. Specifically, the sentences were divided into four versions (A, B, C, D) based on the frequency of foveal words and parafoveal words, with 8 participants in each group, totaling 32 participants. The evaluation results indicated that all sentences demonstrated high fluency, with no significant difference across the four conditions. No participant completed more than one norming task.

To prevent participants from seeing more than one version of the same sentence, the 80 experimental items (8 conditions per item) were distributed into eight experimental blocks using a Latin-square design. Each block contained exactly one condition from each item, ensuring that each participant read each sentence only once and that all eight conditions were equally represented across participants. Each block additionally included 40 filler sentences and 6 practice trials, resulting in 126 trials per participant. Approximately 30% of the trials were followed by a yes/no comprehension question to ensure attentive reading. Participants were randomly assigned to one of the eight blocks.

### Apparatus and procedure

The experimental apparatus used in this study was the EyeLink 1000 plus eye-tracking device, which operates at a sampling rate of 1000 Hz. The display monitor used featured a refresh rate of 144 Hz. The device achieved a viewing angle of approximately 1.12 degrees per character.

Upon arrival at the laboratory, the participants were asked to read and sign the consent form before proceeding with the study. Subsequently, the participant was directed to position their chin on the chin rest, and the experimenter explained the instructions to the participants. The experiment involved participants reading a series of sentences, with the space bar being pressed to advance to the following sentence. After some sentences, there will be a question regarding the semantic understanding of the just-read sentence, to which participants will respond with a “yes” or “no” keypress. A total of 36 such questions will be presented. Prior to the commencement of the formal test, participants were allowed to familiarise themselves with the procedure. The formal experiment involved a three-point calibration procedure every four sentences and lasted approximately 30 minutes.

## Results

The accuracy rate of reading judgments was 95.79%, indicating thorough comprehension of the sentences. Initially, any fixations exceeding 1200 ms or falling below 80 ms were excluded, and (1) A total of 0.55% of the trials were excluded due to head movements. Although participants were instructed before the experiment to maintain head stability by keeping their chins on the chinrest, occasional movements, such as minor posture adjustments, could still occur during the reading task. In such cases, we carefully recorded these trials in real time and excluded them to ensure data accuracy; (2) 1.39% of the trials were excluded due to gaze points on sentences equal to or fewer than 5; and (3) based on the boundary paradigm outlier deletion criteria, trials were excluded for (a) delayed boundary changes (approximately 18.91% of the total); (b) premature boundary changes (about 0.61% of the total); (c) boundary changes by eye “hooks” (approximately 5.86% of the total); (d) boundary changes triggered by saccade (about 0.27% of the total); (e) boundary changes triggered by blinking (about 1.19% of the total). In total, 28.77% of trials were deleted, leaving 71.23% for analysis.

The eye movement measures and statistical analysis methods are referenced to those described by Sun et al. (2024). The data were analysed using Linear Mixed Models (LMMs), with the lme4 package for data processing. Continuous data were log-transformed. Foveal and parafoveal word frequency and preview types, as well as two-way and three-way interactions, were included as fixed effects in the analysis. High-frequency words were coded as level 1, while low-frequency words were coded as level 2. Statistical power was evaluated using simulation-based methods applied to the fitted linear mixed-effects models, as recommended for crossed designs that incorporate both subject- and item-level random variance. Using the simr package in R (200 simulations; α = .05), we estimated the power to detect the theoretically critical interaction between foveal word frequency and preview type. Moreover, the eye-movement measures were primarily concerned with first-pass reading, including temporal metrics such as first fixation duration (FFD), single fixation duration (SFD), gaze duration (GD), and spatial metrics such as skipping probability (SP) and forward saccade length (FSL).

### Analysis of the foveal words

The results demonstrated a word frequency effect of the foveal words, with shorter fixation durations and a higher SP for high-frequency words compared to low-frequency words (SP: *z* = −4.17, *p* < 0.001; Fixation durations: *t*s > 5.94, *p*s < 0.001). The main effect of parafoveal word frequency was found to be insignificant for both SP and all fixation durations (SP: *z* = 1.16, *p* = 0.25; Fixation durations: |*t*s| < 1.08, *p*s > 0.05), which was similar to the non-significant main effect of preview type (SP: *z* = 0.50, *p* = 0.62; Fixation durations: |*t*s| < 0.94, *p*s > 0.05). However, a significant interaction between foveal and parafoveal word frequency was observed for SFD, suggesting the emergence of a parafoveal-on-foveal (PoF) effect associated with word frequency (refer to S1 Fig). More specifically, when the foveal word is of high frequency, its processing time remains unchanged regardless of the parafoveal word frequency. In contrast, when the foveal word is of low frequency, its processing time is affected by the frequency of the parafoveal word, with shorter processing times for foveal words when the parafoveal word is of high frequency (249 ms) compared to low frequency (256 ms). No other significant interactions were identified.

Furthermore, the main effect of all three factors was found to be statistically significant for FSL (|*t*s| > 2.74, *p*s < 0.01). Moreover, the frequency of parafoveal words interacted with the preview type to influence FSL (*t* = 4.03, *p* < 0.001). Specifically, when the preview was identical, the forward saccade length for high-frequency parafoveal words was greater than that for low-frequency words. However, when the preview was pseudocharacters, no significant difference in forward saccade lengths was observed between high- and low-frequency parafoveal words. Other two- and three-way interactions were insignificant, suggesting that processing in both the fovea and parafovea independently influenced the forward saccade length. Further details can be found in Tables 2 and 3.

**Table 2:** Eye movement measures on the foveal and parafoveal region.

Foveal word	High-frequency				Low-frequency			
Parafoveal word	High-frequency		Low-frequency		High-frequency		Low-frequency	
Preview type	Identical	Pseudocha.	Identical	Pseudocha.	Identical	Pseudocha.	Identical	Pseudocha.
Foveal word								
SP	0.20 (.40)	0.20 (.40)	0.22 (.42)	0.20 (.40)	0.15 (.35)	0.15 (.36)	0.15 (.35)	0.18 (.38)
FFD	237 (86)	238 (84)	240 (83)	232 (80)	252 (96)	252 (96)	260 (90)	255 (93)
SFD	236 (86)	239 (86)	238 (81)	229 (74)	249 (94)	249 (94)	261 (90)	252 (90)
GD	246 (99)	264 (121)	264 (125)	254 (127)	288 (143)	297 (180)	293 (138)	306 (177)
FSL	1.96 (.81)	1.58 (.88)	1.76 (.74)	1.52 (.56)	1.91 (.93)	1.54 (.70)	1.71 (.83)	1.48 (.54)
Parafoveal word								
SP	0.27 (.44)	0.10 (.30)	0.18 (.39)	0.09 (.29)	0.24 (.43)	0.10 (.30)	0.18 (.39)	0.07 (.25)
FFD	244 (88)	290 (109)	255 (95)	294 (112)	254 (92)	287 (120)	255 (105)	290 (127)
SFD	243 (89)	303 (111)	252 (91)	307 (108)	250 (90)	293 (121)	255 (101)	296 (128)
GD	280 (145)	351 (144)	287 (137)	372 (171)	287 (155)	333 (154)	303 (164)	357 (183)

“Pseudocha.” stands for “Pseudocharacter” The fixation durations is measured in ms and the FSL is in characters.

**Table 3:** Fixed effects estimates from the linear mixed-effects models at the foveal word.

Fixed effect	*b*	CI	*SE*	*t/z*	*p*
Skipping probability					
Foveal word frequency	**−0.39**	**[-0.57,-0.22]**	**0.09**	**−4.46**	**<0.001**
Parafoveal word frequency	−0.05	[-0.27,0.16]	0.11	−0.48	0.633
Preview type	0.13	[-0.08,0.34]	0.11	1.24	0.217
Foveal word frequency×Parafoveal word frequency	0.18	[-0.03,0.40]	0.11	1.71	0.087
Foveal word frequency×Preview type	0.31	[-0.12,0.73]	0.22	1.41	0.160
Parafoveal word frequency×Preview type	0.30	[-0.12,0.72]	0.21	1.41	0.161
Foveal word frequency×Parafoveal word frequency×Preview type	−0.01	[-0.43,0.41]	0.22	−0.03	0.975
First fixation duration					
Foveal word frequency	**0.07**	**[0.05,0.09]**	**0.01**	**6.22**	**<0.001**
Parafoveal word frequency	0.01	[-0.01,0.03]	0.01	0.81	0.419
Preview type	−0.01	[-0.03,0.01]	0.01	−0.67	0.505
Foveal word frequency×Parafoveal word frequency	0.03	[-0.02,0.07]	0.02	1.24	0.216
Foveal word frequency×Preview type	0.00	[-0.04,0.05]	0.02	0.10	0.924
Parafoveal word frequency×Preview type	−0.03	[-0.07,0.02]	0.02	−1.24	0.217
Foveal word frequency×Parafoveal word frequency×Preview type	0.00	[-0.08,0.09]	0.04	0.07	0.943
Single fixation duration					
Foveal word frequency	**0.07**	**[0.05,0.09]**	**0.01**	**5.95**	**<0.001**
Parafoveal word frequency	0.01	[-0.01,0.04]	0.01	0.83	0.413
Preview type	−0.01	[-0.03,0.01]	0.01	−0.799	0.429
Foveal word frequency×Parafoveal word frequency	**0.05**	**[0.00,0.09]**	**0.02**	**1.99**	**0.047**
Foveal word frequency×Preview type	0.00	[-0.04,0.05]	0.02	0.18	0.861
Parafoveal word frequency×Preview type	−0.04	[-0.09,0.00]	0.02	−1.80	0.072
Foveal word frequency×Parafoveal word frequency×Preview type	−0.01	[-0.10,0.08]	0.05	−0.16	0.876
Gaze duration					
Foveal word frequency	**0.12**	**[0.09,0.15]**	**0.02**	**7.83**	**<0.001**
Parafoveal word frequency	0.01	[-0.01,0.04]	0.01	1.07	0.283
Preview type	0.01	[-0.02,0.04]	0.01	0.93	0.355
Foveal word frequency×Parafoveal word frequency	0.02	[-0.03,0.07]	0.03	0.70	0.482
Foveal word frequency×Preview type	0.01	[-0.04,.0.07]	0.03	0.45	0.657
Parafoveal word frequency×Preview type	−0.04	[-0.10,0.01]	0.03	−1.62	0.105
Foveal word frequency×Parafoveal word frequency×Preview type	0.08	[-0.02,0.19]	0.06	1.53	0.126
Forward saccade length					
Foveal word frequency	**−0.04**	**[-0.06,-0.01]**	**0.01**	**−2.75**	**0.008**
Parafoveal word frequency	**−0.05**	**[-0.07,-0.03]**	**0.01**	**−4.40**	**<0.001**
Preview type	**−0.18**	**[-0.21,-0.15]**	**0.01**	**−12.65**	**<0.001**
Foveal word frequency×Parafoveal word frequency	−0.00	[-0.05,0.04]	0.02	−0.02	0.981
Foveal word frequency×Preview type	0.01	[-0.04,0.05]	0.02	0.39	0.698
Parafoveal word frequency×Preview type	**0.09**	**[0.05,0.14]**	**0.02**	**4.03**	**<0.001**
Foveal word frequency×Parafoveal word frequency×Preview type	−0.01	[-0.10,0.08]	0.05	−0.29	0.774

Significant effects are indicated in bold.

### Analysis of the parafoveal words

**Fig 2:**
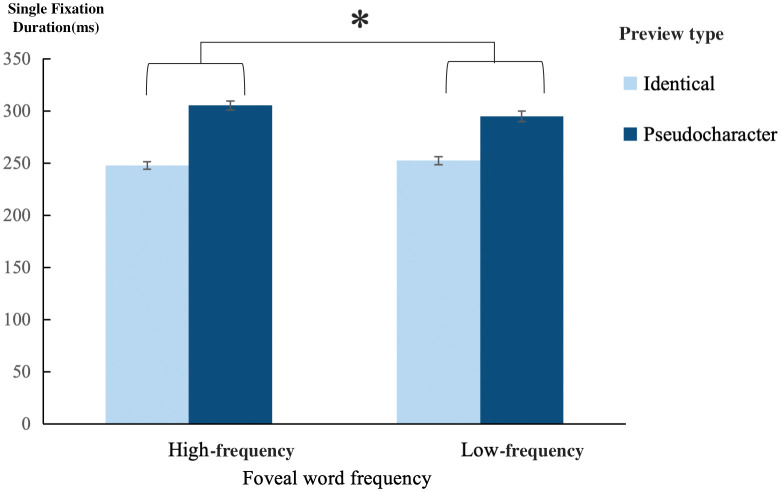
The foveal load effect, exemplified by SFD (* indicates *p* < 0.05).

The main effect of foveal word frequency was insignificant in SP and all fixation durations (SP: *z* = −1.32, *p* = 0.19; Fixation durations: |*t*s| < 1.23, *p*s > 0.05), indicating an absence of spillover effect. The word frequency effect was also observed in the parafoveal word, as demonstrated by significant results in GD (*t* = 2.58, *p* = 0.01) and SP (*z* = −3.39, *p* < 0.001). The main effect of preview type reached statistical significance for SP and all fixation durations (SP: *z* = −11.11, *p* < 0.001; Fixation durations: *t*s > 7.01, *p*s < 0.001), indicating shorter processing times and higher SP when the parafoveal word was identical preview, thereby demonstrating a stable preview effect. Of particular importance, foveal word frequency affects preview benefit, as evidenced by both SFD and GD (|*t*s| > 2.43, *p*s < 0.05). Specifically, a greater preview benefit was observed when the foveal word was of high frequency (SFD: 57 ms; GD: 78 ms) compared to low frequency (SFD: 42 ms; GD: 50 ms), demonstrating the presence of the foveal load effect (refer to Fig 2). A simulation-based power analysis conducted on the fitted linear mixed-effects models showed that the estimated power to detect this foveal word frequency × preview interaction was 73.5% for SFD (95% CI = 66.8–79.5%; *β* = 0.068) and 89.5% for GD (95% CI = 84.4–93.4%; *β* = 0.091). Additionally, parafoveal word frequency did not affect the preview benefit (SP: *z* = 0.88, *p* = 0.38; Fixation durations: |*t*s| < 0.33, *p*s > 0.05). Moreover, the three-way interaction was not observed (SP: *z* = −1.48, *p* = 0.14; Fixation durations: |*t*s| < 0.83, *p*s > 0.05), which suggests that the foveal load effect was not influenced by the parafoveal word frequency. Further details can be found in Tables 2 and 4.

**Table 4:** Fixed effects estimates from the linear mixed-effects models at the parafoveal word.

Fixed effect	*b*	CI	*SE*	*t/z*	*p*
Skipping probability					
Foveal word frequency	−0.09	[-0.33,0.16]	0.13	−0.68	0.500
Parafoveal word frequency	**−0.76**	**[-1.02,-0.50]**	**0.13**	**−5.73**	**<0.001**
Preview type	**−1.46**	**[-1.77,-1.15]**	**0.16**	**−9.15**	**<0.001**
Foveal word frequency×Parafoveal word frequency	**−0.70**	**[-1.03,-0.36]**	**0.17**	**−4.09**	**<0.001**
Foveal word frequency×Preview type	−0.03	[-0.54,0.49]	0.27	−0.10	0.925
Parafoveal word frequency×Preview type	−0.20	[-0.82,0.42]	0.32	−0.64	0.524
Foveal word frequency×Parafoveal word frequency×Preview type	−0.18	[-0.84,0.49]	0.34	−0.52	0.605
First fixation duration					
Foveal word frequency	−0.00	[-0.03,0.02]	0.01	−0.22	0.826
Parafoveal word frequency	0.01	[-0.01,0.04]	0.01	1.18	0.239
Preview type	**0.13**	**[0.10,0.17]**	**0.02**	**7.02**	**<0.001**
Foveal word frequency×Parafoveal word frequency	−0.03	[-0.07,0.02]	0.02	−1.04	0.297
Foveal word frequency×Preview type	−0.03	[-0.08,0.01]	0.02	−1.43	0.154
Parafoveal word frequency×Preview type	−0.00	[-0.05,0.04]	0.02	−0.18	0.861
Foveal word frequency×Parafoveal word frequency×Preview type	0.04	[-0.06,0.13]	0.05	0.82	0.414
Single fixation duration					
Foveal word frequency	−0.02	[-0.04,0.01]	0.01	−1.22	0.222
Parafoveal word frequency	0.02	[-0.00,0.05]	0.01	1.62	0.106
Preview type	**0.17**	**[0.13,0.21]**	**0.02**	**8.57**	**<0.001**
Foveal word frequency×Parafoveal word frequency	−0.01	[-0.07,0.04]	0.03	−0.53	0.597
Foveal word frequency×Preview type	**−0.06**	**[-0.12,-0.01]**	**0.03**	**−2.44**	**0.015**
Parafoveal word frequency×Preview type	−0.01	[-0.06,0.04]	0.03	−0.32	0.750
Foveal word frequency×Parafoveal word frequency×Preview type	0.02	[-0.09,0.12]	0.05	0.29	0.774
Gaze duration					
Foveal word frequency	−0.01	[-0.04,0.02]	0.01	−0.88	0.381
Parafoveal word frequency	**0.05**	**[0.01,0.08]**	**0.02**	**2.58**	**0.012**
Preview type	**0.22**	**[0.19,0.24]**	**0.01**	**14.86**	**<0.001**
Foveal word frequency×Parafoveal word frequency	0.00	[-0.05,0.06]	0.03	0.14	0.888
Foveal word frequency×Preview type	**−0.09**	**[-0.15,-0.03]**	**0.03**	**−3.13**	**0.002**
Parafoveal word frequency×Preview type	0.01	[-0.05,0.06]	0.03	0.28	0.778
Foveal word frequency×Parafoveal word frequency×Preview type	0.01	[-0.10,0.12]	0.06	0.15	0.884

Significant effects are indicated in bold.

### Supplementary analyses

To investigate the impact of foveal load on preview benefit for each character of the two-character parafoveal words, the words were analyzed by dividing them into single-character regions. The main effect of the preview type was observed in all fixation durations of the first character (*t*s > 6.09, *p*s < 0.001). No other significant interactions were identified on the first character. A similar main effect of preview type was observed for the second character (*t*s > 2.49, *p*s < 0.05). Of greater significance, however, was the finding that the frequency of the foveal word influences preview benefit for all fixation durations on the second character (|*t*s| > 2.49, *p*s < 0.05). This finding suggests a foveal load effect on the second character, implying that the influence of foveal load within two-character words is likely to stem from the second character. Further details can be found in Tables 5 and 6.

**Table 5:** Eye movement measures in the supplementary analyses.

Foveal word	High-frequency				Low-frequency			
Parafoveal word	High-frequency		Low-frequency		High-frequency		Low-frequency	
Preview type	Identical	Pseudocha.	Identical	Pseudocha.	Identical	Pseudocha.	Identical	Pseudocha.
First character								
FFD	250 (100)	294 (109)	255 (97)	298 (111)	253 (97)	296 (123)	251 (97)	297 (121)
SFD	250 (101)	297 (111)	256 (98)	303 (111)	251 (94)	295 (122)	249 (93)	301 (124)
GD	258 (110)	312 (120)	260 (102)	311 (115)	256 (103)	309 (136)	262 (122)	317 (134)
Second character								
FFD	243 (79)	271 (105)	253 (96)	274 (117)	249 (86)	262 (115)	259 (108)	258 (124)
SFD	243 (80)	271 (104)	254 (96)	275 (119)	247 (83)	264 (116)	259 (109)	255 (112)
GD	249 (88)	277 (112)	256 (98)	282 (123)	255 (105)	269 (118)	264 (112)	264 (146)

“Pseudocha.” stands for “Pseudocharacter.” The fixation durations is measured in ms.

**Table 6:** Fixed effects estimates from the linear mixed-effects models in the supplementary analyses.

Fixed effect	*b*	CI	*SE*	*t/z*	*p*
First character					
First fixation duration					
Foveal word frequency	0.00	[-0.03,0.03]	0.02	0.04	0.965
Parafoveal word frequency	0.01	[-0.03,0.04]	0.02	0.30	0.765
Preview type	**0.15**	**[0.10,0.19]**	**0.02**	**6.08**	**<0.001**
Foveal word frequency×Parafoveal word frequency	−0.03	[-0.10,0.04]	0.03	−0.89	0.375
Foveal word frequency×Preview type	−0.01	[-0.08,0.05]	0.03	−0.37	0.713
Parafoveal word frequency×Preview type	0.00	[-0.06,0.07]	0.03	0.11	0.916
Foveal word frequency×Parafoveal word frequency×Preview type	0.04	[-0.09,0.17]	0.07	0.65	0.517
Single fixation duration					
Foveal word frequency	0.01	[-0.05,0.03]	0.02	−0.40	0.693
Parafoveal word frequency	0.01	[-0.02,0.05]	0.02	0.75	0.453
Preview type	**0.16**	**[0.13,0.20]**	**0.02**	**9.48**	**<0.001**
Foveal word frequency×Parafoveal word frequency	−0.02	[-0.09,0.04]	0.03	−0.71	0.478
Foveal word frequency×Preview type	−0.02	[-0.08,0.05]	0.03	−0.50	0.618
Parafoveal word frequency×Preview type	0.01	[-0.06,0.08]	0.03	0.29	0.772
Foveal word frequency×Parafoveal word frequency×Preview type	0.04	[-0.10,0.17]	0.07	0.53	0.599
Gaze duration					
Foveal word frequency	−0.00	[-0.04,0.04]	0.02	−0.01	0.989
Parafoveal word frequency	0.01	[-0.02,0.05]	0.02	0.77	0.441
Preview type	**0.18**	**[0.13,0.23]**	**0.02**	**7.37**	**<0.001**
Foveal word frequency×Parafoveal word frequency	−0.00	[-0.07,0.07]	0.03	−0.00	0.997
Foveal word frequency×Preview type	−0.01	[-0.08,-0.06]	0.03	−0.29	0.772
Parafoveal word frequency×Preview type	0.01	[-0.06,0.08]	0.04	0.24	0.808
Foveal word frequency×Parafoveal word frequency×Preview type	0.05	[-0.08,0.19]	0.07	0.75	0.455
Second character					
First fixation duration					
Foveal word frequency	−0.02	[-0.05,0.02]	0.02	−0.96	0.335
Parafoveal word frequency	0.01	[-0.02,0.04]	0.02	0.60	0.548
Preview type	**0.04**	**[0.01,0.08]**	**0.02**	**2.49**	**0.016**
Foveal word frequency×Parafoveal word frequency	−0.02	[-0.09,0.04]	0.03	−0.71	0.477
Foveal word frequency×Preview type	**−0.08**	**[-0.14,-0.01]**	**0.03**	**−2.40**	**0.017**
Parafoveal word frequency×Preview type	−0.03	[-0.09,0.04]	0.03	−0.84	0.403
Foveal word frequency×Parafoveal word frequency×Preview type	−0.01	[-0.14,0.12]	0.07	−0.15	0.878
Single fixation duration					
Foveal word frequency	−0.02	[-0.05,0.01]	0.02	−1.15	0.252
Parafoveal word frequency	0.01	[-0.02,0.04]	0.02	0.55	0.586
Preview type	**0.05**	**[0.01,0.08]**	**0.02**	**2.62**	**0.011**
Foveal word frequency×Parafoveal word frequency	−0.03	[-0.09,0.04]	0.03	−0.82	0.414
Foveal word frequency×Preview type	**−0.07**	**[-0.14,-0.01]**	**0.03**	**−2.24**	**0.025**
Parafoveal word frequency×Preview type	−0.04	[-0.10,0.03]	0.03	−1.15	0.250
Foveal word frequency×Parafoveal word frequency×Preview type	−0.02	[-0.15,0.11]	0.07	−0.25	0.804
Gaze duration					
Foveal word frequency	−0.02	[-0.05,0.01]	0.02	−1.13	0.259
Parafoveal word frequency	0.01	[-0.03,0.04]	0.02	0.38	0.705
Preview type	**0.05**	**[0.01,0.09]**	**0.02**	**2.70**	**0.009**
Foveal word frequency×Parafoveal word frequency	−0.03	[-0.09,0.04]	0.03	−0.79	0.428
Foveal word frequency×Preview type	**−0.08**	**[-0.15,-0.02]**	**0.03**	**−2.45**	**0.015**
Parafoveal word frequency×Preview type	−0.03	[-0.10,0.04]	0.03	−0.83	0.409
Foveal word frequency×Parafoveal word frequency×Preview type	−0.03	[-0.17,0.10]	0.07	−0.51	0.609

Significant effects are indicated in bold.

## Discussion

This study explored the effect of foveal and parafoveal word frequency on preview benefit during Chinese reading. The skipping rate of foveal words was 18%, which supports the reliability of the preview data. Additionally, the stable frequency effect observed for both foveal and parafoveal words, along with the preview effect, suggests that the experimental manipulations were effective.

### The effect of foveal word frequency on preview

The results demonstrated that when parafoveal words consist of two characters, foveal word frequency exerts an impact on preview benefit. Specifically, as the foveal load increases, the preview benefit decreases, indicating the presence of the foveal load effect. These findings align with previous studies that used two-character words as parafoveal words [35] and differ from studies where the parafoveal word was a single character [25,26,34]. Importantly, simulation-based power analyses further support the robustness of the observed foveal word frequency × preview interaction. The current experimental design yielded relatively high statistical sensitivity for gaze duration (GD; power = 89.5%). Although the power estimate for single fixation duration (SFD) was comparatively lower (73.5%), it nonetheless falls within a moderately high range and allows for cautious interpretation of this measure. The corresponding effect sizes were small to moderate (GD: *β* = 0.091; SFD: *β* = 0.068). Taken together, these results suggest that the observed interaction pattern is unlikely to be solely attributable to insufficient statistical power. Accordingly, conclusions based on GD—and to a more qualified extent those based on SFD—should be interpreted as reasonably reliable under the present experimental design.

As previously mentioned, the primary eye movement control models for alphabetic languages simulate the foveal load effect. The E-Z Reader model posits that words are processed sequentially. After the familiarity checks for the foveal word (L1 stage), saccade planning occurs, and after lexical access (L2 stage), attention begins to shift, resulting in the processing of the parafoveal word. Therefore, the more challenging the foveal word is, the longer lexical access takes, extending the L2 stage and delaying attention shifts. Given the fixed duration of saccade planning, the time available for parafoveal processing is reduced, which results in a diminished preview benefit [12,13]. This mechanism has been extended to Chinese reading in the Chinese E-Z Reader model, which assumes presegmented word boundaries and applies the same serial lexical processing stages, successfully simulating the foveal load effect in Chinese [40]. In contrast, the SWIFT model, which represents the Guidance of Attention Gradient (GAG) model, suggests that attention is distributed in parallel within the perceptual span, allowing readers to process words simultaneously. According to this model, it can be inferred that more difficult foveal words take longer to process, providing more time for the adjacent parafoveal word and resulting in a greater preview benefit [14–16]. This suggests that the SWIFT model’s prediction contrasts with that of the E-Z Reader model.

Although the Chinese extension of the E-Z Reader model has demonstrated that its core assumptions can account for the foveal load effect in Chinese reading when word boundaries are presegmented by the experimenter [40], the model does not explicitly incorporate word segmentation processes in its architecture. Consequently, recent work has emphasized that an adequate account of Chinese eye-movement control must integrate competitive word segmentation mechanisms operating on unspaced character strings [33].

Drawing on Chinese characteristics, Li and Pollatsek (2020) put forward the CRM grounded in the interactive activation model. The CRM posits that characters within the perceptual span are processed in parallel. All possible words formed from the activated characters are simultaneously activated, and words that overlap in spatial position compete with each other, with the unique winner completing both word recognition and word segmentation. Word recognition proceeds sequentially until all words in the sentence are identified. When a word’s activation level exceeds a threshold of 0.3, its word frequency begins to influence the preview processing of subsequent words, with the preview benefit increasing as the foveal word frequency rises. Consequently, the CRM’s predictions regarding the foveal load effect align with those of the E-Z Reader model.

Additionally, parafoveal processing is deeper because of the Chinese’s distinct features—particularly its compact character arrangement. Consequently, this study observed a PoF effect of word frequency on SFD, aligned with previous studies [41]. In our data, this PoF effect emerged only in SFD, suggesting that competition among potential word candidates may influence early stages of lexical encoding. This pattern is more compatible with the Chinese Reading Model, which assumes parallel activation of multiple position-specific word candidates within the perceptual span. Under this framework, competing word candidates are activated early and simultaneously, and their competition outcomes determine both word segmentation and subsequent eye-movement control dynamics. Because SFD is particularly sensitive to early lexical processing, parafoveal lexical properties can exert an influence on foveal processing at this stage, naturally giving rise to a PoF effect. In contrast, such early competitive interactions are not explicitly implemented in the E-Z Reader model.

Therefore, considering both the direction of the foveal load effect and the presence of the PoF effect on SFD, the current results align more closely with the CRM. Nevertheless, the CRM still faces challenges in explaining inconsistencies in the foveal load effect between single- and two-character parafoveal words, indicating that further refinement of the model is necessary.

### The effect of parafoveal word frequency on preview

The findings also revealed that parafoveal word frequency has no effect on the preview benefit, consistent with Wang et al. (2019) while differing from patterns observed in alphabetic languages [10,19].

The results suggest that parafoveal word frequency affects both the skipping probability and fixation duration (GD) of parafoveal words, as well as a PoF effect of word frequency observed on the SFD. These findings suggest that the information acquired during parafoveal processing activates a certain level of lexical processing. However, parafoveal word frequency has no effect on the preview benefit, possibly due to incomplete word recognition. In contrast to alphabetic languages, Chinese readers need additional resources for word segmentation, which could explain the differences in how parafoveal word frequency affects preview processing between Chinese and alphabetic languages.

Additionally, the results help clarify whether parafoveal processing occurs in word-based units. Firstly, a PoF effect based on word frequency was observed, confirming the role of words in parafoveal processing. Secondly, parafoveal word frequency influenced parafoveal words’ fixation duration (GD), suggesting that word-based processing contributes to preview processing. Therefore, these findings offer evidence that parafoveal processing takes place at the level of individual words.

### The effect of foveal and parafoveal word frequency on preview

The study did not find a combined effect of foveal and parafoveal word frequency on preview, which is consistent with the findings of Sun et al. (2024) while differing from the findings observed in alphabetic languages. Taken together with previous discussions on the effects of foveal and parafoveal word frequency on preview, these results suggest that the preview benefit is solely related to the processing difficulty of the foveal words, not parafoveal words. This is more in line with the serial processing view, which posits that foveal processing consumes more attentional resources, leaving fewer resources available for parafoveal processing. As a result, the allocation of attentional resources to parafoveal processing is determined by the difficulty of foveal processing. Although the CRM asserts that character processing within the perceptual span occurs parallel, word processing is sequential, with word recognition and segmentation occurring simultaneously. Therefore, this finding also aligns with the CRM.

**Fig 3:**
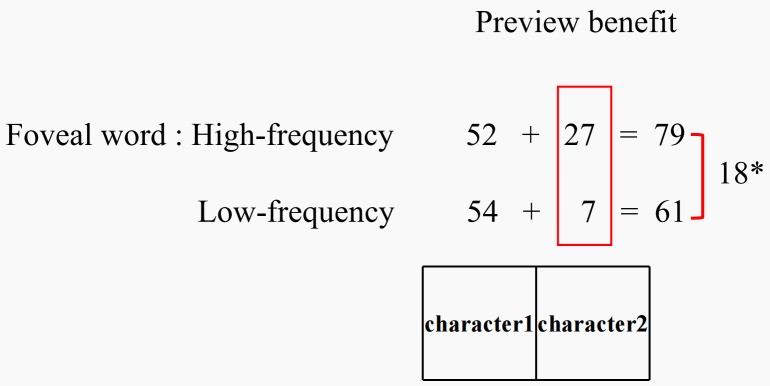
Preview benefit for the two single-character spaces under different foveal loads (using GD as an example).

Furthermore, the absence of evidence for a three-way interaction indicates that the foveal load effect was not influenced by parafoveal word frequency. Consequently, the observed difference in the foveal load effect between single- and two-character words in the parafoveal region is likely attributable to the intrinsic properties of word length rather than parafoveal processing difficulty. A supplementary analysis investigated how parafoveal word length influences the foveal load effect, dividing the parafoveal two-character word into two regions: the first and second characters. The results implied that the foveal load effect on the preview benefit originated primarily from the second character of the two-character word. According to the CRM framework, readers process Chinese characters in parallel within the perceptual span, with processing efficiency being influenced by both the distance from the fovea and visual attention. As the distance from the fixation point increases, recognition efficiency decreases. Therefore, one possible speculation is that the degree to which processing efficiency is reduced due to decreased visual acuity may vary under different foveal loads. No difference in preview benefit is observed in the area closest to the fovea, but such differences may become apparent in regions farther from the fovea. These supplementary analyses suggest that the foveal load effect may represent a spatial “accumulation” of preview benefit differences across word lengths. For example, in the GD measure, the preview benefit for the first character was 52 ms under high-frequency conditions (foveal load) and 54 ms under low-frequency conditions, showing no statistically significant difference. However, a notable difference was observed for the second character: the preview benefit was 27 ms under high-frequency conditions and 7 ms under low-frequency conditions. This pattern closely mirrored that observed for the entire two-character word, where the overall preview benefit was 79 ms and 61 ms, respectively (see Fig 3). This hypothesis requires further validation in subsequent experiments.

Regarding forward saccade length, the results show that both foveal and parafoveal processing loads independently influence the length of the forward saccade. Specifically, when the foveal processing load is lower, the forward saccade length increases, and when more parafoveal preview information is available, the forward saccade length also becomes longer. This finding aligns with the predictions of the CRM, which employs a strategy based on processing efficiency to simulate saccadic target selection. According to this model, readers aim to process as much information as possible during each fixation. When processing efficiency at a given fixation drops below a certain threshold, the eyes shift to an unprocessed location. Thus, the CRM suggests that the greater the information acquired to the right of fixation, the longer the forward saccade will be [33].

### Limitations and summary

This study has several limitations. First, the present study did not manipulate word length, and therefore our interpretation regarding the potential influence of word length on the foveal load effect remains tentative. The current findings demonstrate that a foveal load effect occurs when both foveal and parafoveal words are two-character words. Whether such effects depend on word length requires future research that systematically varies character length at both foveal and parafoveal positions. Accordingly, the present results should be regarded as complementary to prior studies reporting null foveal load effects using single-character words, rather than as direct evidence that word length determines the emergence of foveal load effects in Chinese reading. Second, the hypothesis that the foveal load effect represents a spatial “accumulation” effect is relies on the assumption that the parafoveal word is either of very high or low frequency and that the two-character word space is artificially divided into the first and second characters. This is a preliminary approach and may not fully capture the complexities of the foveal load effect. Future studies should explore the foveal load effect more comprehensively within the parafoveal range. Third, the effects of foveal and parafoveal word frequency on preview may be asymmetric. While foveal word frequency influences both identical and pseudocharacter preview, parafoveal word frequency affects only identical preview. As a result, the impact of foveal load on pseudocharacter preview may introduce confounding effects. However, in this study, using orthographically legal pseudocharacters as the baseline minimizes the processing cost associated with invalid preview. Consequently, the impact of foveal load on the preview of orthographically legal pseudocharacters is negligible and does not affect the results.

This study investigated whether the processing load of foveal and parafoveal words or their combined effect influences parafoveal preview during Chinese reading, using two-character words in both foveal and parafoveal positions. The experimental manipulation was effective, yielding the following key findings:

First, foveal word frequency significantly affected preview benefit, with higher foveal word frequency leading to a larger preview benefit. This finding supports the presence of a foveal load effect and aligns with the predictions of the CRM. However, the results for two-character parafoveal words differed from those for single-character words and could not be easily explained by existing models.

Second, parafoveal word frequency had no effect on the preview benefit. However, it did affect the processing time and skipping rate of parafoveal words, and a PoF effect on word frequency was observed. These findings suggest that parafoveal processing may achieve a certain level of lexical activation without reaching complete lexical identification.

Third, no interaction was observed between foveal and parafoveal word frequency in predicting preview benefit, indicating that parafoveal word frequency does not impact the foveal load effect. The discrepancy in the foveal load effect between single- and two-character parafoveal words was not attributable to the greater processing difficulty of two-character words but rather to differences in word length. Further analysis revealed that the foveal load effect for two-character words primarily stemmed from the second character, suggesting that the effect may result from an accumulation of preview benefit differences across word length.

Finally, the study found that foveal and parafoveal processing loads independently influenced forward saccade length. Specifically, lower processing load in both regions led to longer forward saccades, consistent with the CRM predictions.

## Conclusions

The study demonstrates that when parafoveal words consist of two characters, foveal word frequency influences preview benefit, thereby confirming the presence of the foveal load effect. In contrast, no evidence suggests that parafoveal word frequency has any impact. Additionally, no combined effect of foveal and parafoveal word frequency on preview. The foveal load effect may indicate a spatial accumulation of differences in preview benefit. These findings support the CRM and provide valuable empirical evidence for its refinement.

## Supporting information

S1 Fig The PoF effect, exemplified by SFD (* indicates p < 0.05). (TIF)
