# Supplementary figures and images for: Further investigation of the impact of foveal and parafoveal word frequency on parafoveal preview during Chinese reading
Source: PLoS One. 2026 Jan 20;21(1):e0340103. doi: 10.1371/journal.pone.0340103 (PMC12818676; doi:10.1371/journal.pone.0340103)

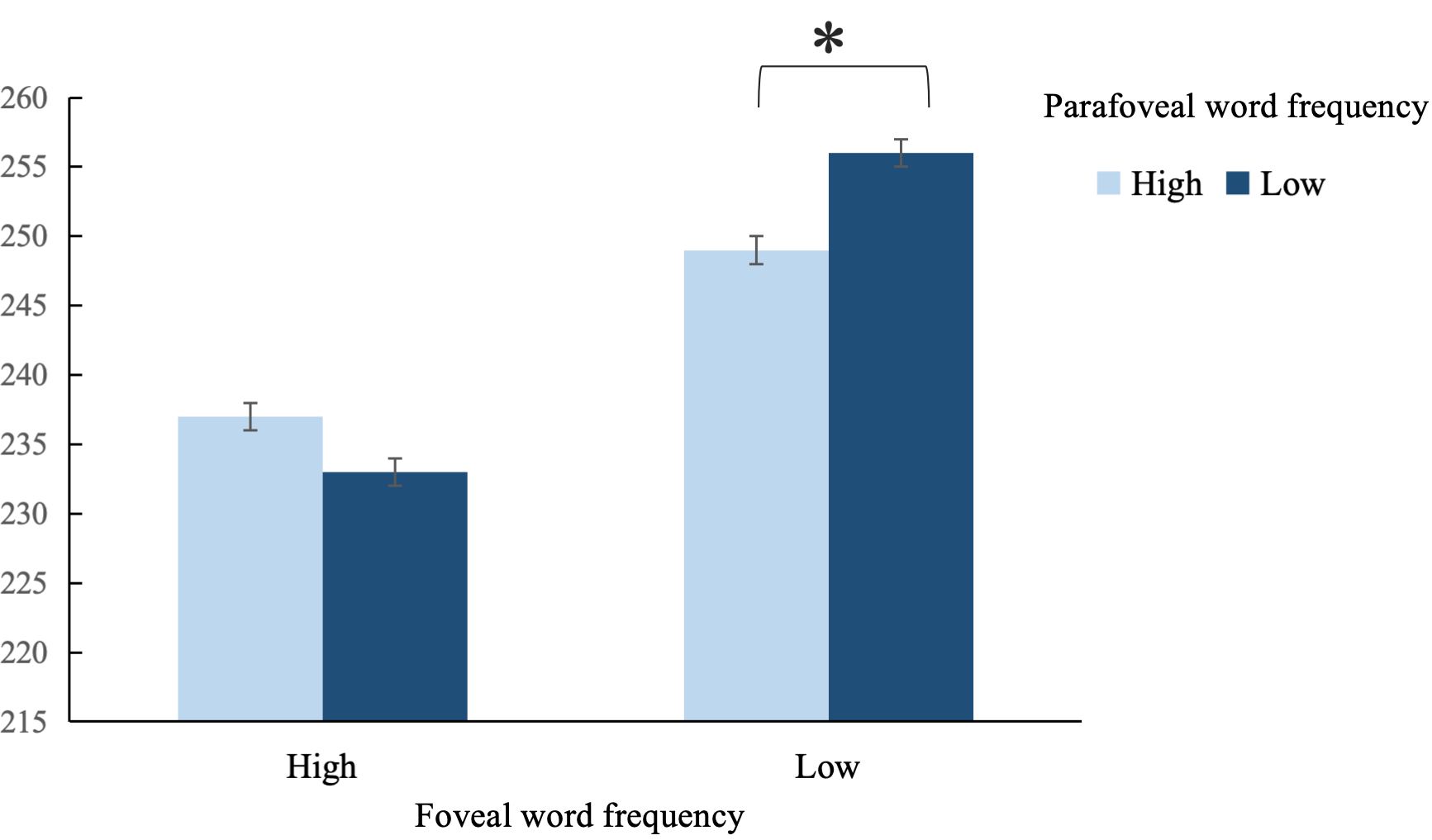

Supplement: S1 Fig — (TIF) [file pone.0340103.s001.tif]
